# Gut and obesity/metabolic disease: Focus on microbiota metabolites

**DOI:** 10.1002/mco2.171

**Published:** 2022-09-01

**Authors:** Ke Lin, Lixin Zhu, Li Yang

**Affiliations:** ^1^ State Key Laboratory of Biotherapy and Cancer Center/Collaborative Innovation Center for Biotherapy West China Hospital Sichuan University Chengdu China; ^2^ Guangdong Institute of Gastroenterology Guangdong Provincial Key Laboratory of Colorectal and Pelvic Floor Disease Sixth Affiliated Hospital of Sun Yat‐Sen University Guangzhou China; ^3^ Department of Colorectal Surgery Sixth Affiliated Hospital Sun Yat‐Sen University Guangzhou China

**Keywords:** gut microbiota, metabolic disease, metabolites, obesity

## Abstract

Obesity is often associated with the risk of chronic inflammation and other metabolic diseases, such as diabetes, cardiovascular disease, and cancer. The composition and activity of the gut microbiota play an important role in this process, affecting a range of physiological processes, such as nutrient absorption and energy metabolism. The active gut microbiota can produce a large number of physiologically active substances during the process of intestinal metabolism and reproduction, including short‐chain/long‐chain fatty acids, secondary bile acids, and tryptophan metabolites with beneficial effects on metabolism, as well as negative metabolites, including trimethylamine N‐oxide, delta‐valerobetaine, and imidazole propionate. How gut microbiota specifically affect and participate in metabolic and immune activities, especially the metabolites directly produced by gut microbiota, has attracted extensive attention. So far, some animal and human studies have shown that gut microbiota metabolites are correlated with host obesity, energy metabolism, and inflammation. Some pathways and mechanisms are slowly being discovered. Here, we will focus on the important metabolites of gut microbiota (beneficial and negative), and review their roles and mechanisms in obesity and related metabolic diseases, hoping to provide a new perspective for the treatment and remission of obesity and other metabolic diseases from the perspective of metabolites.

## Graphical abstract

The beneficial and negative metabolites produced by gut microbiota. The gut microbiota produces all kinds of beneficial or negative metabolites and directly affects the intestinal barrier and gut hormone (GLP‐1 and PYY) release. After entering circulation, those metabolites regulate satiety, insulin release, adipose tissue function, and systemic immunity, thus influencing obesity and energy metabolism.

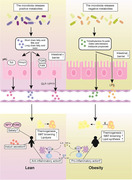

## 1 INTRODUCTION

Obesity is a major global health challenge, and its health risks have attracted wide attention. Obesity is not only manifested in changes in appearance but is also associated with disrupted lipid and glucose metabolism, chronic inflammation, and an increased risk of multiple metabolic diseases, such as diabetes, cardiovascular disease, and cancer. Although many factors contribute to the development of obesity/metabolic diseases, various lines of evidence link these diseases to changes in the composition and activity of the gut microbiome.^1^ The gut microbiota includes up to 100 trillion symbiotic microbes that live in the gut, relying on food scraps, intestinal mucus, and more.^2^ The normal gut microbiome of the human body is mainly composed of *Firmicutes*, *Bacteroides*, *Proteus*, *Actinomycetes*, and *Fusobacteria*, in which *Firmicutes* and *Bacteroides* dominate. Some of the main functions of gut microbiota include: (1) resistance to pathogen infection through direct competition for nutrients and attachment sites; (2) affecting intestinal barrier permeability; (3) regulating intestinal immunity; (4) obtaining energy from indigestible diets; and (5) secreting metabolites with different physiological activities. Therefore, changes in the structure and metabolism of gut microbiota affect the host in many ways, including energy absorption, metabolism, and even immunity.

In the context of obesity, the potential of gut microbiota metabolites to contribute to energy absorption and metabolism is interesting. Obesity may be associated with two dominant phyla: *Firmicutes* and *Bacteroidetes*. Turnbaugh et al.^3^ found that obese mice had a higher relative abundance of *Firmicutes* in their microbiota and an enhanced ability of gut microbiota to derive energy from diet, the trait that fecal microbiome transplantation (FMT) results suggested could be transferred. Similarly, the gut microbiota of obese people has this signature.^4^ Recent studies have linked obesity to a variety of specific bacteria, such as *Akkermansia muciniphila*, *Bifidobacteria*, and *Lactobacillus*. Several independent trials have shown that supplementation with *Akkermansia* significantly improves metabolism, improves insulin sensitivity, and reduces plasma triglycerides.^5^, ^6^ Similarly, *Lactobacillus* and *Bifidobacteria* also play an important role in the balance of human intestinal microecology. For example, *Bifidobacteria* was used to treat obese youth, and insulin sensitivity was significantly improved.^7^ How the gut microbiota specifically affects and participates in metabolic and immune activities, especially the metabolites directly produced by the gut microbiota, has attracted extensive attention.

In fact, active gut microbiota can secrete a mass of physiological active substances, including short‐chain fatty acids (SCFAs) and secondary bile acids (BAs), in the process of intestinal metabolism and reproduction. These metabolites can enter the whole body blood circulation through the intestinal tract and directly or indirectly affect human metabolism and immune processes. A metagenomic association study and serum metabonomic analysis found that the abundance of *Bacteroides thetaiotaomicron* in obese individuals decreased significantly and was negatively correlated with serum glutamate concentration. Intragastric administration reduced plasma glutamate concentrations and reduced diet‐induced weight gain and obesity in mice.^8^ Another research has shown that people with type 2 diabetes (T2D) were characterized by dysbiosis of gut microbiota, a decrease in the abundance of butyrate‐producing bacteria and an increase in various opportunistic pathogens.^9^ Selectively increasing the diversity and abundance of SCFA‐producing bacteria increases host glucagon‐like peptide 1 (GLP‐1) production and decreases metabolically harmful compounds.^10^ This evidence suggests that metabolites secreted by microbiota in intestinal tract are key factors in host–microbiota interactions.

**FIGURE 1:**
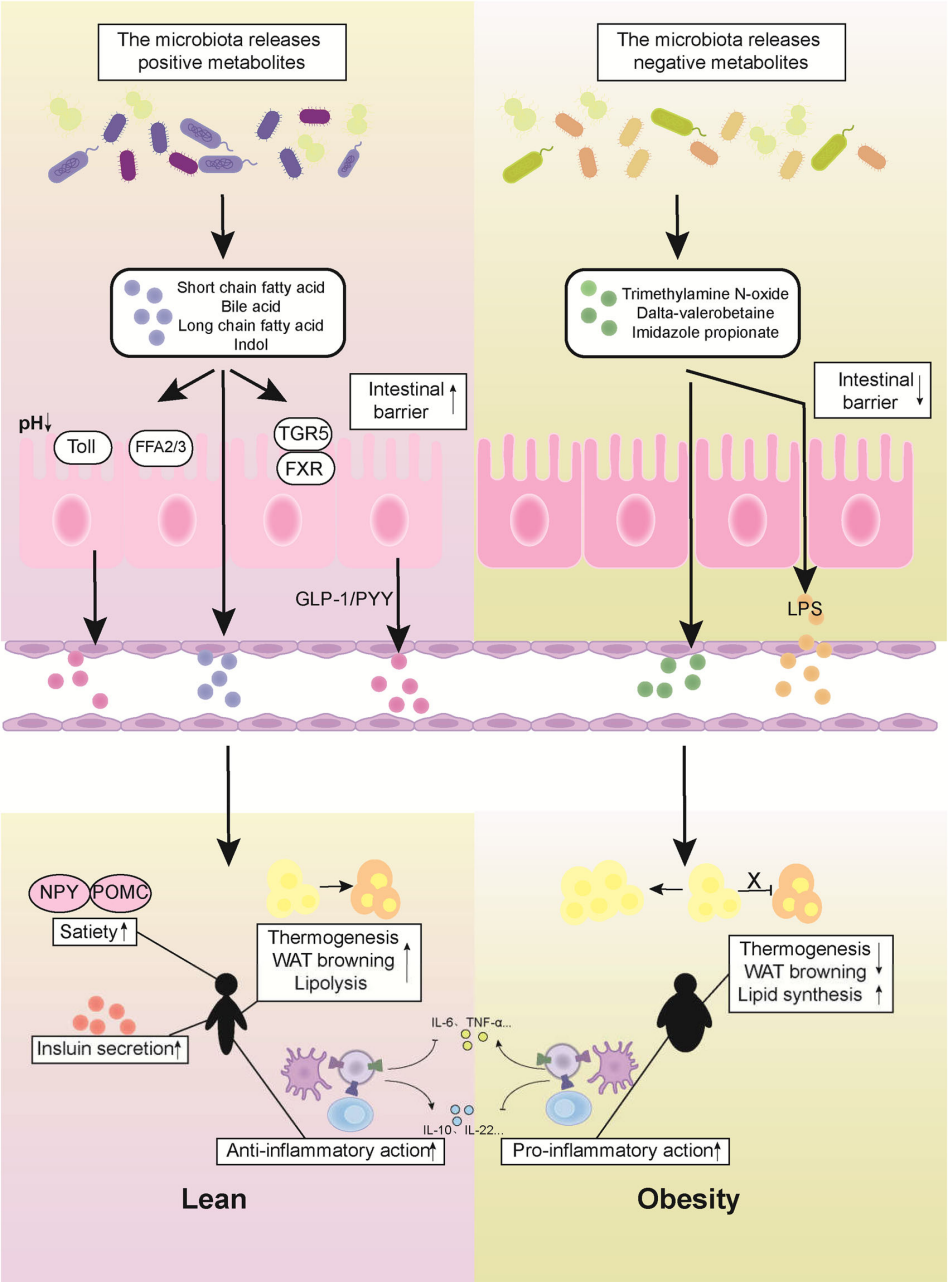
The beneficial and negative metabolites produced by gut microbiota. The gut microbiota produces all kinds of beneficial or negative metabolites and directly affects the intestinal barrier and gut hormone (glucagon‐like peptide 1 [GLP‐1] and tyrosine tyrosine [PYY]) release. After entering circulation, those metabolites regulate satiety, insulin release, adipose tissue function, and systemic immunity, thus influencing obesity and energy metabolism.

Considering the influence of gut microbiota on the occurrence, development, and consequences of obesity and other disease, understanding gut microbiota and its metabolites is helpful to the treatment of obesity. Several studies have shown that gut microbiota metabolites are associated with a variety of host metabolic characteristics, such as obesity, diabetes, and other metabolic diseases. The occurrence and progression of these diseases often involve energy intake, insulin secretion, changes in adipose tissue function, and systemic chronic inflammation. Some previous studies have also proved that gut microbiota metabolites can affect obesity characteristics through these mechanisms. In this review, we will state beneficial and negative metabolites produced by gut microbiota and their mechanisms to affect obesity and related metabolic diseases (Figure 1).

## 2 BENEFICIAL METABOLITES OF GUT MICROBIOTA

Gut microbiota produces a variety of metabolites with physiological effects in the intestinal tract, which are absorbed into the host circulation and can be detected in the blood, brain, and other tissues. SCFAs and secondary BAs, which are beneficial metabolites, are the focus of recent studies. Sufficient human and animal studies have proved that SCFAs and BAs are associated with obesity and metabolic disorders, and they are involved in the processes of nutrient intake, insulin secretion, and immunity to affect the characteristics of obesity in hosts. However, indoles and long‐chain fatty acids (LCFAs) are rarely studied, and most of them only prove their correlation with obesity characteristics, waiting for further research on the mechanism. The intermediate substance of mutual crosstalk between intestinal flora and host. This section introduces four important metabolites of gut microbiota that are thought to be beneficial.

### 2.1 Short‐chain fatty acids

SCFAs is one of the most studied and common metabolites of gut microbiota, generally including butyrate, propionate, and acetate. It is produced by intestinal bacterial fermentation of undigested food components. After being absorbed by colon cells, these SCFAs are used locally as an energy source for the epithelial cells of the colon mucous membrane or enter the portal vein bloodstream for circulation. Absorption of SCFAs in the gut: diffusion of undissociated forms and active transport of dissociated forms by SCFAs transporters. There are two types of SCFAs transporters: monocarboxylate transporters (MCT1/MCT4)^11^ and sodium‐coupled monocarboxylate transporters (SMCT1/SMCT2) that are coupled with a transmembrane H^+^ gradient.^12^ MCT1, SMCT1, and SMCT2 transporters are expressed in the apical membrane of the intestinal epithelium, while MCT1 and MCT4 are expressed in the basolateral region. Transporter expression is regulated by SCFAs and inflammation, and the uptake of SCFAs may be altered in obesity and certain disease states.^13^, ^14^ Therefore, there may be bidirectional regulation between obesity or other diseases and SCFA levels.

Acetate is the most abundant SCFAs in the colon, accounting for more than half of the total SCFAs detected in feces.^15^ There are two main ways to produce acetate: through fermentation or synthesis of acetate through the Wood–Ljungdahl pathway.^16^ Propionate is synthesized in three ways: the succinate pathway (*Bacteroidetes* and some *Firmicutes* bacteria), acrylate pathway (*Veillonellaceae*, *Coprococcus catus*, etc.), and the propanediol pathway (members of the *Proteobacteria* and *Lachnospiraceae* families).^17^ Two different butyrate production pathways are known: the butyrate kinase pathway and the butyryl‐CoA:acetate CoA‐transferase pathway.^18^ In addition, some bacteria do not produce SCFAs themselves, such as *Lactobacillus* and *Bifidobacterium*, and their decomposition of dietary fiber provides the necessary substrates (i.e., oligosaccharides, lactic acid, and acetate) for use by other SCFA‐producing bacteria.^19^, ^20^

Clinical studies found that the total amount of SCFAs in obese subjects was significantly higher than that in lean subjects.^21^ The number of SCFA‐producing bacteria and SCFAs decreased in fecal samples from metabolically abnormal mice, as well as from obese and diabetic patients. The composition of the gut microbiota in mice treated with alternate‐day fasting changed, and the levels of acetate and lactic acid in serum increased.^22^ An increase in total SCFAs level may indicate an increased ability of obese individuals to extract calories from otherwise indigestible foods. An increase in acetate levels could mean it is metabolically beneficial or that the microbes that produce acetic acid are more likely to increase in this way.

After metformin treatment, T2D patients showed increased fecal potential for butyrate production.^23^ Another study further demonstrated an increase in serum acetate and butyrate after 6 months of metformin use.^24^ Treatment with a variety of probiotics (*Lactobacillus plantarum*, *Lactobacillus bulgaricus*, *Lactobacillus gasseri*, etc.) in combination with metformin showed increased plasma butyrate concentration and enrichment of microbial butyrate production pathways.^25^ An increase in serum acetate was associated with a decrease in fasting insulin,^24^, ^26^ and increased butyrate production in the gut was associated with improved insulin sensitivity.^27^, ^28^ Moreover, supplementation with SCFAs can improve metabolism by increasing energy expenditure, glucose tolerance, and homeostasis in diabetic and obese animals.^10^, ^29^, ^30^ In humans, SCFAs stimulate the production of GLP‐1 and tyrosine tyrosine (PYY), leading to weight loss.^31^ In normal male mice, studies of Lep^ob/ob^ and PPAR‐γ^Lox/Lox^ mice demonstrated that butyrate can improve metabolism and reduce obesity by promoting GLP‐1 and PYY release and fatty acid oxidation and enhancing mitochondrial function.^32^, ^33^, ^34^

However, another study in pregnant female Sprague–Dawley (SD) rats found that butyrate was associated with increased intramural fat deposition and insulin resistance in offspring.^35^ Although the effect of butyrate on metabolism is still controversial, SCFAs represented by butyrate are thought to improve obesity and metabolism in general.

### 2.2 Bile acids

BAs are produced in the liver as a water‐soluble, cholesterol‐derived amphiphilic molecule of saturated hydroxylated C‐24 sterols. Rate‐limiting enzymes are CYP7A1 (the classical pathway) and sterol‐27‐hydroxylase (CYP27A1) (the alternative pathway).^36^ The main BAs produced in humans are cholic acid (CA) and chenodeoxycholic acid (CDCA), while the main BAs produced in rodents are CA and muricholic acid (MCA), especially β‐MCA, at the primary level. After BAs are combined with taurine (mostly in mice) or glycine (mostly in humans), the primary BAs are secreted from the liver into bile, and food is ingested into the duodenum. In distal ileum intestinal cells, 95% of BAs are reabsorbed by the apical sodium‐dependent bile acid transporter (ASBT/SLC10A2) through enterohepatic circulation (EHC). Transport back to the liver, another 5% of BAs are eliminated through feces.^37^

The gut microbiota converts primary BAs into secondary BAs through the following main reactions.^38^ Bile salt hydrolases (BSHs) cleave the amide bond between the glycine and taurine moieties conjugated to the steroid nucleus of bile salts to liberate BAs, which is the crucial first step for further BAs alterations by microbes within the intestinal environment. BSHs are represented in bacteria, including *L* *actobacillus*, *Bifidobacterium*, *Enterococcus*, *Clostridium* spp, est.^39^ Conjugated BAs in the gut are known to be toxic to bacteria, especially at low pH, and affect bacterial growth in different areas of the gastrointestinal tract (GIT). As a result, the presence of BSHs can exert a protective effect on certain bacterial species.^40^ After that, bile salts were liberated by BSHs, and further metabolism by microbiota began. These BA modifications are performed mainly by anaerobes in the lower intestine and include reamidation, oxidation–reduction reactions, and esterification and desulfation reactions.^41^ Bacteria with 7α‐dehydroxylation activity form deoxycholic acid (DCA) and lithocholic acid (LCA) in the human intestine by CA and CDCA, respectively, while αMCA and βMCA form murideoxycholic acid (MDCA) in the mouse intestine. This process occurs mainly in *Clostridium firmicutes* or *Eubacters*.^42^ Bacteria can also undergo oxidation, epimerization, and dihydroxylation through hydroxysteroid dehydrogenases (HSDHs).^39^, ^43^ Secondary BAs showed strong antibacterial activity and cytotoxicity.

Many studies have documented changes in plasma BAs levels in metabolic diseases such as obesity and diabetes.^44^, ^45^ Because BAs are a key ligand for the Takeda G‐protein‐coupled receptor 5 (TGR5) and the farnesoid X receptor (FXR), which regulate glucose and lipid metabolism, signaling affects many different metabolic processes in the host, TGR5 is activated primarily by the secondary BAs LCA and DCA, while the main ligand for FXR is CDCA, followed by CA, DCA, and LCA. BAs change significantly in patients with metabolic diseases such as obesity, and insulin resistance is associated with an increase in total.^46^ The level of ursodeoxycholic acid (UDCA) in EHC of high‐fat diet (HFD) rats decreased, while the level of DCA increased, accompanied by an increase in *Coprococcus*, *Lactococcus*, *Blautia*, and *Ruminococcus*.^47^ In obesity‐resistant mice, secondary BAs, including UDCA, CDCA, and LCA, were reduced and were associated with changes in the gut microbiota, such as increases in *Akk. muciniphila* and *uc_Atopobiaceae*. The correlation found that *C* *lostridium scindens* and *Clostridium hylemonae* were positively correlated with UDCA, LCA, and DCA.^48^ In addition, feeding UDCA and LCA significantly improved fasting glucose, insulin resistance, and obesity in HFD mice.^48^, ^49^ An increase in serum BAs means that fewer BAs are excreted in the stool. Some studies proved this: patients undergoing bariatric surgery with Roux‐en‐Y gastric bypass (RYGB) had reduced fecal levels of primary and secondary BAs, such as CDCA, CA, LCA, DCA, and UDCA, possibly due to BSH activity from intestinal bacteria, such as *Blautia* and *Veillonella*. Its absorption and recycling are increased through the enterohepatic pathway.^50^, ^51^ In addition, the CA/CDCA ratio was increased in the early feces of RYGB recipients.^50^ Another study showed an increased circulating CA/CDCA ratio in pregnant women with intrahepatic cholestasis, which is associated with increased fasting triglycerides and elevated blood sugar.^52^ This indicates that the circulating CA/CDCA ratio is closely related to blood glucose and metabolism.

Some probiotics have the ability to regulate BAs metabolism. In vitro evidence confirmed that Parabacteroides distasonis can convert primary BAs to secondary BAs (LCA and UDCA), activate FXR‐fibroblast growth factor 15 (FGF15), and reduce weight gain, hyperglycemia, and hepatic steatosis in ob/ob and HFD mice.^49^ Primary BAs (CA and CDCA) decreased and secondary BAs (DCA and LCA) increased after *Lactobacillus acidophilus* was fed to mice with high cholesterol.^53^ After feeding *A. muciniphila* and quercetin, total plasma BAs increased, and secondary BAs increased overall, especially ωMCA and UDCA. The combination therapy reduced the levels of fasting blood glucose and plasma insulin and prevented obesity and inflammation.^54^ In addition, the CA/(glychocholic acid (GCA) + taurocholic acid (TCA) ratio was positively correlated with *A. muciniphila*, which may be related to BSHs.^54^ Lactis 420™ (B420) was administered alone and in combination with prebiotics. The results showed changes in microbial and plasma BAs. Treatment increased *Christensenellaceae* spp., *Bifidobacterium S24‐7*, *Barnesiellaceae* spp., *A. muciniphila*, *Streptococcus*, and *Lactobacillus* and was accompanied by reductions in plasma BAs, including glycocholic acid, glycoursodeoxycholic acid, taurohyodeoxycholic acid, and tauroursodeoxycholic acid (TUDCA).^55^

### 2.3 Long‐chain fatty acids

LCFAs are saturated and/or unsaturated fatty acids containing 14–20 carbons and play an important role in regulating energy metabolism. Recent studies have shown that the gut microbiota *Enterococcus faecalis* can drive the production of LCFAs (specifically myristoleic acid) through ACOT genes. HFD mice supplemented with *E. faecalis* showed weight loss, increased energy metabolism, and brown adipose tissue (BAT) activation.^56^ In another study, *Escherichia coli Nissle 1917* (EcN) and *Holdemanella biformis* were shown to produce 3‐hydroxy‐octadecenoic acid (C18‐3OH), which is involved in the in vivo anti‐inflammatory process and excites peroxisome proliferator‐activated receptor γ (PPAR‐γ).^57^ At present, there are few studies on LCFAs and gut microbiota, and LCFAs are mainly ingested from food.^58^ Therefore, the role of LCFAs derived from the gut microbiota in obesity and metabolic diseases needs further research.

### 2.4 Metabolites of tryptophan

Tryptophan is an essential amino acid found in milk, cheese, fish, and other foods.^59^ Tryptophan is a precursor to the synthesis of many important biologically active molecules, such as serotonin, melatonin, niacinamide, and vitamin B3, among many other important substances. Tryptophan is metabolized through three pathways: the serotonin, kynurenine, and indole pathways.

Most dietary tryptophan is metabolized through the kynurenine pathway, through the rate‐binding enzyme indoleamine 2,3‐dioxygenase 1 (IDO1) in immune cells and intestinal epithelial cells, or to NAD^+^ in the liver by tryptophan 2,3‐dioxygenase (TDO). Part of tryptophan is synthesized into 5‐hydroxytryptamine (5‐HT) in intestinal chromaffin cells. In addition, approximately 5% of tryptophan is metabolized in the intestinal tract by the bacterial tryptophanase of the gut microbiota into a series of indole metabolites, such as indole‐3‐aldehyde (IAld), indole‐3‐acid‐acetic (IAA), indole‐3‐propionic acid (IPA), and indole‐3‐lactic acid (ILA).^60^

Clinical studies found that the levels of IAA, ILA, and IPA were significantly reduced in obese subjects.^61^ Among them, IPA and IAA are the most important indole metabolites. IPA reduces the risk of T2D and has been shown to be associated with gut microbiota diversity.^62^ Similarly, mice fed with HFD had lower levels of IPA and IAA in their serum.^63^, ^64^ A higher IPA concentration was negatively correlated with the incidence of diabetes but positively correlated with higher dietary fiber intake, suggesting that gut microbiota may play an important role.^65^ Indole metabolites, including IAA, ILA, and IPA, were negatively correlated with 5‐HT and kynurenine/tryptophan levels in obese people. This suggests that the metabolism of tryptophan through the gut microbiota pathway is decreased, while the kynurenine and serotonin pathways are increased under the influence of obesity.^61^

**FIGURE 2:**
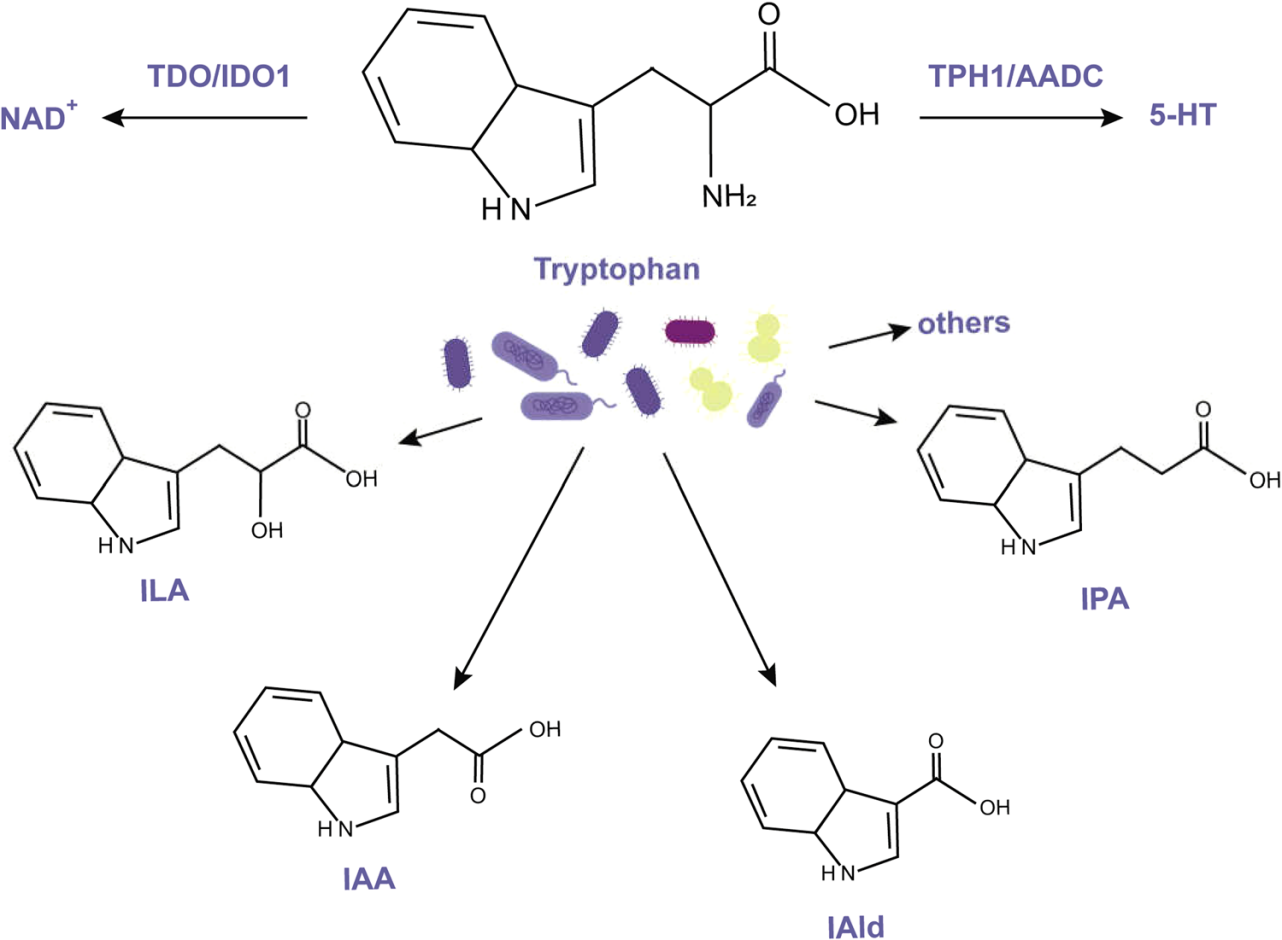
Tryptophan metabolism. Most dietary tryptophan is metabolized to NAD+ in the liver. Part of tryptophan is synthesized into 5‐hydroxytryptamine (5‐HT). Approximately 5% of tryptophan is metabolized in the intestinal tract by the bacterial tryptophanase of the gut microbiota into a series of indole metabolites, such as indole‐3‐aldehyde (IAld), indole‐3‐acid‐acetic (IAA), indole‐3‐propionic acid (IPA), and indole‐3‐lactic acid (ILA).

Tryptophan catabolism bacteria that produce indole derivatives include anaerobes, *Bacteroides*, *Clostridium*, *Bifidobacterium*, and *Lactobacillus*. Tryptophan and indole active transporters have been demonstrated in *E. coli*, *Lactobacillus reuteri*, and *Lactobacillus johnsonii* catalyze tryptophan catabolism to IAld by the enzyme aromatic amino acid aminotransferase.^66^ However, because the indole pathway of tryptophan requires a series of enzymes and different bacteria have different metabolic enzymes, it is difficult to accurately describe the enzymatic pathway of bacterial indole metabolite production.^67^ Probiotics such as *Akkermansia*, *Lactobacillus*, and *Bifidobacteria* can affect tryptophan metabolism.^68^ In a recent study, *A. muciniphila*, either live or pasteurized, reduced elevated serum levels of tyrosine, phenylalanine, and tryptophan metabolites in diabetic patients.^69^ The intestinal flora of mice fed with a HFD changed, the abundance of *Bacteroidetes* and *Akkermansia* decreased, and the serum IAA level decreased.^63^ However, how *Akkermansia* reduces the tryptophan metabolite level remains to be further explored. Another study of the oral administration of *Bifidobacterium longeum BL21* in male C57BL/6J mice suggests that probiotics may affect tryptophan metabolism by balancing the gut microbiota (Figure 2).^70^, ^71^

## 3 NEGATIVE METABOLITES OF GUT MICROBIOTA

This section introduces three negative metabolites: trimethylamine N‐oxide (TMAO), delta‐valerobetaine (VB), and imidazole propionate (ImP). TMAO is an important metabolite among them, which has been proved to be related to host metabolic disorders and obesity, and it has been verified that it can indeed play a role in the development of obesity. At present, there are few studies on these negative metabolites, which are mainly related to systemic chronic inflammation and adipose tissue function changes, and other mechanisms need to be further explored. But what is debatable is whether these metabolites are markers of impaired metabolism, the result of impaired metabolism, or both?

### 3.1 Trimethylamine N‐oxide

TMAO is a trimethylamine (TMA) metabolized from nutrients in food (such as phosphatidylcholine, choline, etc.) by intestinal microbial enzymes (such as CutC/D and CntA/B), which are then further metabolized by the host enzyme flavin‐containing monooxygenase 3 (FMO3) in the liver.^72^ Choline catabolism increases the level of circulating TMO.

In several separate human and animal studies, TMAO has been shown to increase obesity, T2D, and insulin resistance.^73^, ^74^, ^75^, ^76^, ^77^ It has been found that a HFD impairing mitochondrial function in the colonic epithelium enhances the enteric bioavailability of oxygen and nitrate, thereby enhancing the respiration‐dependent choline catabolism of *E. coli*.^78^ After weight loss and RYGB, plasma TMAO levels decrease, along with obesity and insulin resistance.^79^, ^80^ Dietary interventions in obese and glucose‐intolerant patients can reduce TMAO levels and regulate postprandial glucose levels.^81^

Currently, TMA‐producing bacteria are reported as obligate anaerobic *Clostridia* (phylum *Firmicutes*) and facultatively anaerobic *Enterobacteriaceae* (phylum *Proteobacteria*).^82^, ^83^ A new microbial enzyme, CntA/B, has been found to convert carnitine to TMA, a gene present in only a few species, including *E. coli*, *Klebsiella* spp., and *Citrobacter* spp.^84^ Treatment of mice with the inhibitor iodimethylcholine to inhibit the TMA lyase CutC in gut microbiota and thereby reduce plasma TMAO levels showed effective inhibition of diet‐induced obesity and altered the expression of lipid metabolism genes in white adipose tissue (WAT).^85^ In addition, the choline analog 3,3‐dimethyl‐1‐butanol and the carnitine analog mildronate have also been shown to reduce TMAO levels.^86^ Moreover, TMAO inhibited hepatic BAs synthesis.^87^

### 3.2 Delta‐valerobetaine

VB is a microbial source of diet‐dependent obesogen. It was found to reduce carnitine levels and inhibit mitochondrial fatty acid oxidation in mice.^88^ Because carnitine regulates glucose and lipid metabolism,^89^ VB is the substrate for carnitine transporter (OCTN2). Therefore, VB may reduce the efficiency of carnitine reuptake, that is, increase the elimination rate of urinary carnitine.

To determine whether the gut microbiota produces VB, the cecal contents of both normal and germ‐free mice were cultured in vitro. The results showed that only normal cecal contents produced VB. It was found that *E. coli (K12)* and *Salmonella typhimurium* produced VB in isolated cultures. Furthermore, VB is one of the precursors of TMA.^88^ This indicated that VB may combine with TMA/TMAO to enhance metabolic disorders and cause obesity.

### 3.3 Imidazole propionate

ImP is a metabolite produced by gut microbiota using histidine and is found at higher levels in the blood of T2D and pre‐T2D patients. This means that ImP is associated with impaired glucose metabolism, not with diabetic status.^90^ Analysis of gut microbiota in T2D patients showed that ImP levels were associated with a low abundance of microbial diversity and *Bacteroidetes*. Another study also demonstrated that ImP can predict the α diversity of human gut microbiota.^91^ ImP increases systemic inflammation and is negatively associated with circulating mucosal‐associated invariant T (MAIT). An unhealthy diet (not histidine intake) alters ImP levels, so it is possible that diet alters gut flora and thus ImP levels. In other words, ImP levels may be a symptom of metabolic impairment and contribute to disease development.

## 4 MECHANISMS AFFECTING OBESITY/METABOLIC DISEASE

Obesity is often associated with an imbalance in energy intake and expenditure, insulin resistance, and chronic inflammation. As previously stated, intestinal flora regulates a variety of host physiological functions, such as energy absorption, intestinal barrier permeability, secondary BA synthesis, and intestinal hormone release. After its metabolites enter the circulation, they can affect appetite through the blood–brain barrier, and affect adipose tissue synthesis, decomposition, thermogenesis, and browning. At the same time, the influence of intestinal flora on intestinal permeability is also associated with systemic chronic inflammation, which is a further factor leading to insulin resistance.^92^ Several mechanisms of intestinal microbiota metabolites affecting obesity have been proposed.

### 4.1 Affect host satiety and energy intake

An important factor causing obesity is that the body absorbs more energy than it consumes, and the excess energy is stored in the form of fat. Food intake and digestion are controlled by the interaction between the GIT and the brain. Gut hormone (GLP‐1, PYY, etc.) and neural signals from the GIT are key participants in this bidirectional signaling pathway.

Gut hormones secreted by L cells, such as GLP‐1 and PYY, are involved in the regulation of food intake, intestinal motility, and insulin secretion. SCFAs, BAs, and indoles produced by gut microbiota can affect the release of gut hormones through different pathways. Peripheral BAs activate TGR5 in intestinal L cells to stimulate the release of GLP‐1, thereby improving glucose tolerance and obesity.^93^ FXR is another important target of BAs. Treatment with the BA‐sequestering resin sevelamer increased GLP‐1 secretion^94^ and improved blood glucose in ob/ob mice in an FXR‐dependent manner, confirming that the FXR/GLP‐1 pathway is a novel mechanism by which BAs regulate glucose metabolism.^95^ SCFAs stimulate the release of GLP‐1 and PYY from intestinal L cells via the G protein‐coupled free fatty acid receptors FFAR2 (GPR43) and FFAR3 (GPR41).^96^, ^97^ In addition, butyrate can also increase PYY expression by upregulating Toll‐like receptors.^98^ PYY affects appetite and satiety by inhibiting neuropeptide Y (NPY) and activating proopiomelanocortin (POMC) neurons in the arcuate nucleus of the hypothalamus (ARC) or by delaying gastric emptying. GLP‐1 indoles have been shown to stimulate GLP‐1 production in intestinal endocrine L cells.^99^ In vitro experiments have shown that indoles inhibit voltage‐gated K^+^ channels, increase the time width of action potential stimulated by L cells, and lead to enhanced Ca^2+^ entry, which dramatically stimulates GLP‐1 secretion.^100^

**FIGURE 3:**
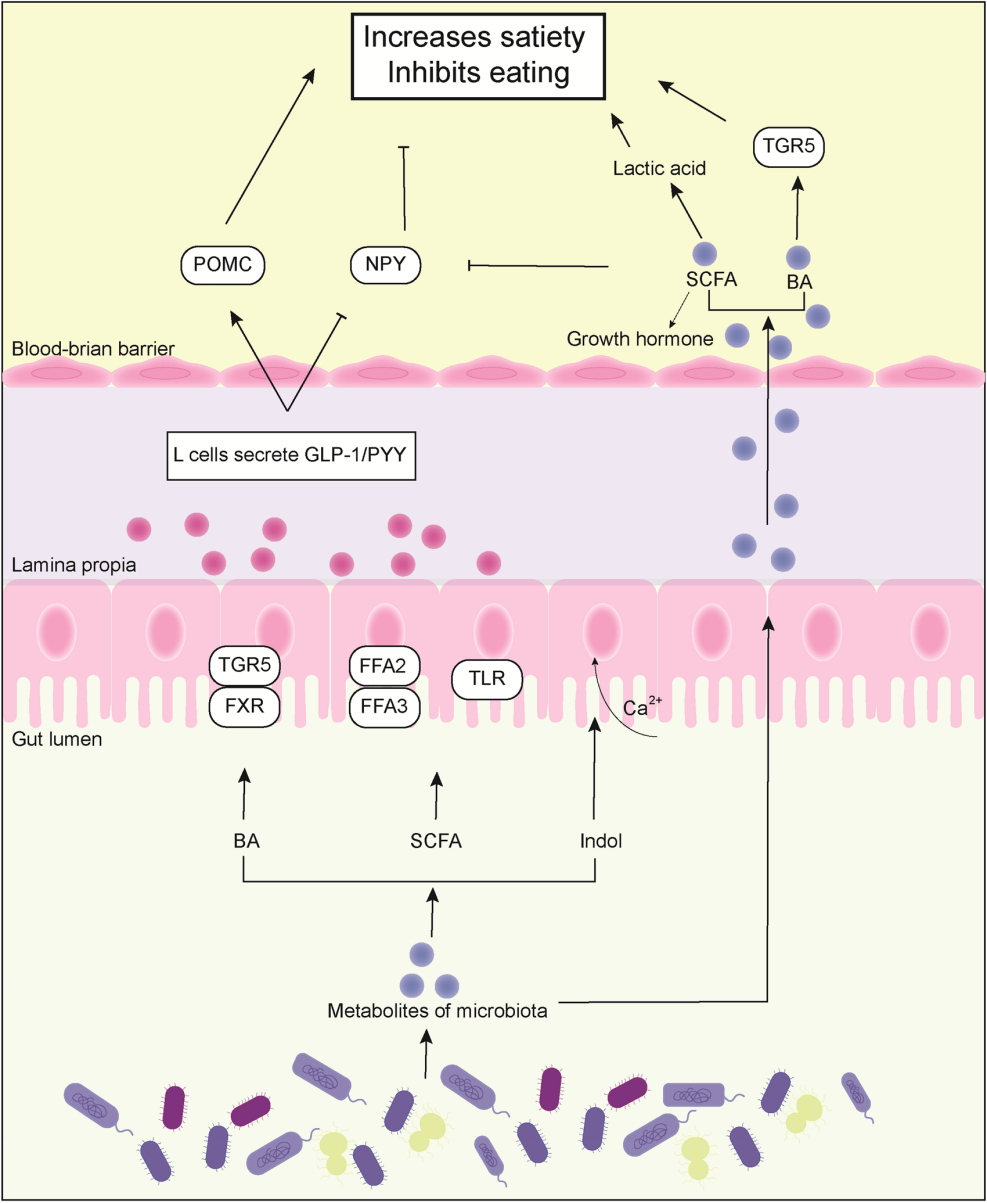
Metabolites enhance satiety and inhibit eating. Through bile acid (BA) receptors (Takeda G‐protein‐coupled receptor 5 [TGR5] and farnesoid X receptor [FXR]), short‐chain fatty acid (SCFA) receptors (GPR41/43), Toll‐like receptor (TLR) of intestinal cells, and enhanced Ca^2+^ entry, L cells can promote the release of intestinal hormones (glucagon‐like peptide 1 [GLP‐1]/tyrosine tyrosine [PYY]), activate hypothalamus proopiomelanocortin (POMC) and inhibit neuropeptide Y (NPY) to improve satiety. Meanwhile, BAs and SCFAs can cross the blood–brain barrier, and BAs enhance satiety through TGR5 receptors in the hypothalamus. SCFAs increase lactic acid and inhibit NPY. Otherwise, SCFAs promote the level of growth hormone, which can control energy homeostasis by stimulating lipolysis and protein retention.

In addition to directly affecting gut hormone production, gut microbiota metabolites can also suppress appetite through the central nervous system. TGR5 was found to be expressed in neurons in the hypothalamus. BAs are also found in the brain, where levels correlate with levels in the circulation. Thus, a recent study revealed a long‐term BA‐dependent hypothalamic mechanism that contributes to weight regulation in obesity. BAs have been shown to improve diet‐related obesity through TGR5 signal transduction in the hypothalamus, including the regulation of food intake and energy expenditure.^101^ Similarly, colonic acetate has been shown to cross the blood–brain barrier to reach the hypothalamus, where it increases the glial circulation of glutamate‐glutamine and gamma‐aminobutyric acid (GABA), increasing lactate levels and thereby suppressing appetite.^102^ In addition, oral butyrate inhibited the activity of appetite‐stimulating neurons expressing NPY in the hypothalamus, decreased the activity of neurons in the solitarytract nucleus and dorsal vagus nerve complex in the brain stem, and ultimately indirectly interfered with the appetite and feeding behavior of the host (Figure 3).^103^

### 4.2 Affect islet β cells and insulin secretion

Islet β cells play an important role in the development of obesity and diabetes by producing and storing large amounts of insulin, which is secreted precisely into the circulation when stimulated by glucose. SCFAs and BAs promote glucose‐stimulated insulin secretion (GSIS) through several pathways.

First, SCFAs may contribute to the β cell response to insulin resistance. The SCFA receptor FFA2/3 is expressed in islet β cells. FFA2/3 levels were upregulated in the islets of HFD mice, although there is some controversy.^104^ FFAR2^−/−^ mice fed with HFD showed impaired glucose tolerance and reduced insulin secretion.^105^ One study demonstrated that FFAR2^−/−^ mice were obese on a normal diet, whereas mice that overexpressed FFAR2 maintained a lean phenotype even when fed with HFD. Moreover, both types of mice reared under sterile conditions or after antibiotic treatment had normal phenotypes, suggesting an important role for the gut microbiota.^106^ The mechanism is that SCFAs are activated in combination with FFA2/3. FFA2 regulates GSIS by Gα_q/11_ (primary) and Gα_i/o_, whereas FFA3 inhibits GSIS by Gα _i/o_. In β cells, increased Ca^2+^ triggers insulin secretion.^107^

Second, islet β cells also express the BA receptors TGR5 and FXR. Stimulation of the islets of male mice with TUDCA was found to increase GSIS in a dose‐dependent manner.^108^ In vitro experiments have shown that selective TGR5 ligands lead to increased insulin secretion in a manner dependent on G s/cAMP/Ca^2+^.^93^ This mechanism also relies on protein kinase A (PKA), since inhibition of PKA reduces TUDCA‐promoted GSIS secretion.^108^, ^109^ In addition, BAs can increase Ca^2+^ and promote insulin secretion through FXR expressed by islet β cells.^110^

**FIGURE 4:**
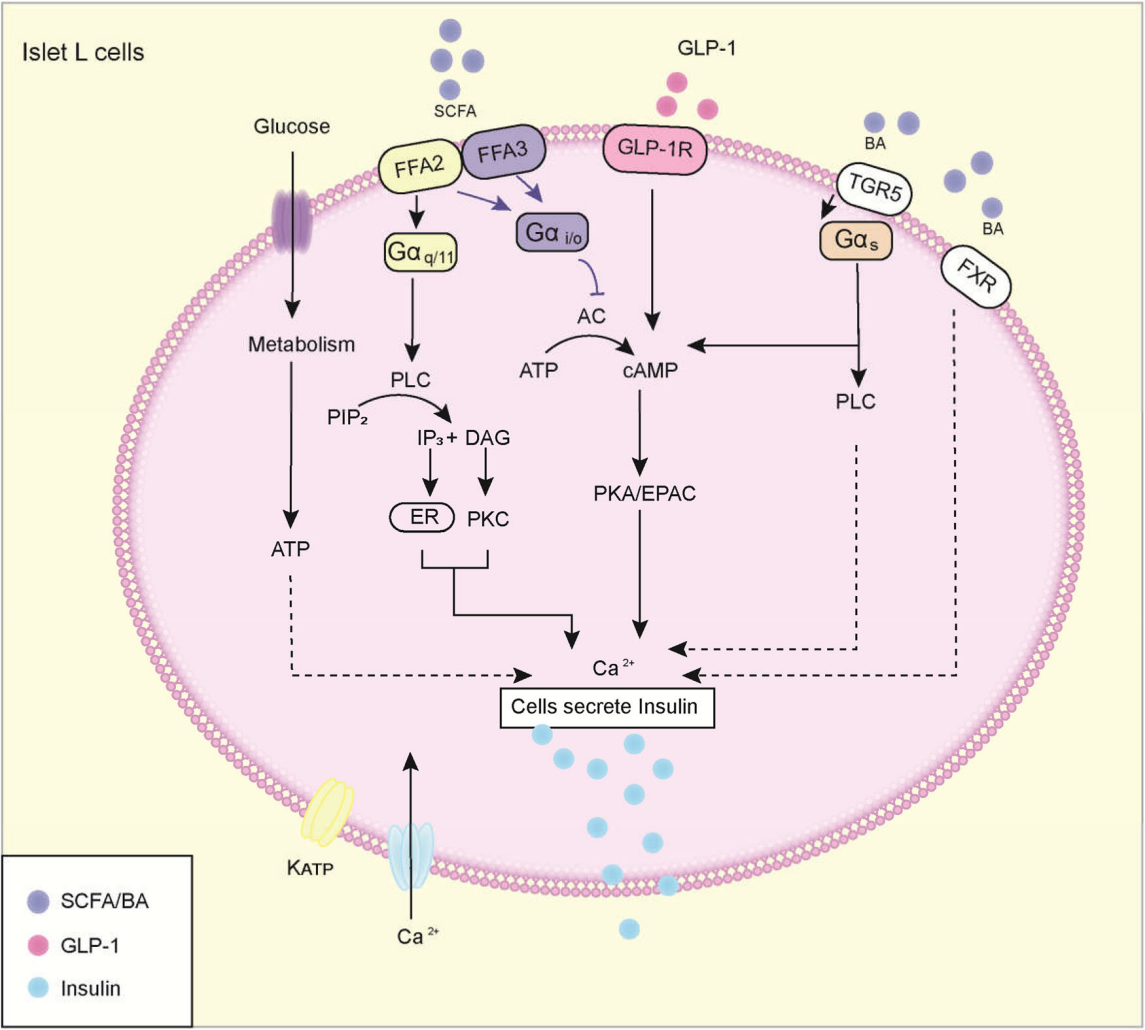
Metabolites affect islet β cells and regulate insulin secretion. When glucose internalized in islet β cells is metabolized, it results in elevation of the adenosine triphosphate (ATP):adenosine diphosphate (ADP) ratio, closure of ATP‐sensitive potassium (KATP) channels, and opening of voltage‐dependent calcium channels (Ca^2+^ channels), causing secretion of insulin. First, short‐chain fatty acids (SCFAs) can improve or decrease glucose‐stimulated insulin secretion (GSIS). After Free fatty acid 2 (FFA2) activation, the Gαq/11 subunit activates phospholipase C (PLC), which hydrolyzes phosphatidylinositol 4,5‐bisphosphate (PIP2) to diacylglycerol (DAG) and inositol triphosphate (IP3), activating protein kinase C (PKC) and releasing calcium ions from endoplasmic reticulum (ER) storage, respectively, which can promote insulin release. FFA2 and FFA3 can also bind to Gα I/O subunits and inhibit adenylate cyclase (AC), thereby reducing the concentration of cyclic AMP (cAMP) and inhibiting protein kinase A (PKA) and exchange protein directly activated by cAMP (EPAC)‐mediated insulin release. Similarly, bile acids (BAs) activate the Takeda G‐protein‐coupled receptor 5 (TGR5) receptor to bind Gα s and promote insulin release through the PLC and cAMP pathways. BAs can also bind to farnesoid X receptor (FXR) receptors to promote Ca^2+^ release. Meanwhile, metabolites (SCFAs, BAs, and indole) promote the release of glucagon‐like peptide 1 (GLP‐1) by intestinal L cells, and its binding with GLP‐1R also promotes the release of insulin.

In addition to acting directly on islet β cells, as mentioned above, the metabolites of gut microbiota BAs, SCFAs, and indole can also promote GSIS indirectly by acting on islet β cells with GLP‐1.^111^ GLP‐1 has the following effects on islets: (1) it stimulates insulin secretion; (2) it increases glucose metabolism by promoting insulin synthesis; and (3) it stimulates β cell proliferation and inhibits apoptosis (Figure 4).^112^

### 4.3 Affect adipose tissue function

Due to adaptive thermogenesis, BAT has become a hot topic in obesity and related metabolic diseases. BAT can metabolize energy substrates to produce heat but does not release chemical energy from adenosine triphosphate (ATP) decomposition. This process is driven by uncoupling protein 1 (UCP‐1). Therefore, increased BAT quality in obese patients may improve metabolism. One possible way to increase the presence of UCP‐1 in adipose tissue is to convert white (pre) adipose cells to brown‐like adipose cells, leading to fat browning.^113^ Both human and mouse studies have proven that BA can induce the expression of UCP‐1 in BAT, increase energy conversion and reduce obesity.^114^, ^115^ A human study showed that BAT activity was negatively correlated with the 12α‐OH/non12α‐OH BA ratio, which may represent the key enzyme CYP8B1 as a target of BAT.^116^ CDCA, LCA, and other TGR5 agonists increase UCP‐1 expression in brown fat cells, suggesting that TGR5 plays an important role in BA‐mediated fat browning.^115^ Activation of TGR5 in BAT increases thermogenesis by converting inactive thyroxine (T4) to active thyroid hormone (T3).^117^ In addition, UDCA was shown to induce a significant elevation of SIRT‐1‐PGC‐1α in adipose tissue.^118^ Myristoleic acid, a type of LCFA from gut microbiota *E. faecalis*, also reduces obesity by activating BAT and forming beige fat.^56^ It has been shown that another LCFA, eicosapentaenoic acid, induces thermogenesis of BAT cells through upregulation of FFAR4‐dependent miR‐30b and miR‐378.^119^ In addition, LCFAs also activate UCP‐1 in fat. Importantly, a recent study showed that LCFAs are essential for UCP‐1 decoupling.^120^ However, the role of intestinal microbiome‐derived LCFAs in obesity and metabolic diseases needs further research. In contrast, TMAO may decrease PGC‐1α expression. When the TMAO‐producing enzyme FMO3 was knocked down, the expression of browning and thermogenic genes such as UCP‐1 was increased. The dietary supply of TMAO reversed the increase in PGC‐1α in WAT driven by knockdown of FMO3.^121^ In addition, TMAO and TMA may be related to insulin signaling in adipocytes, with hormone‐like signaling.^122^

**FIGURE 5:**
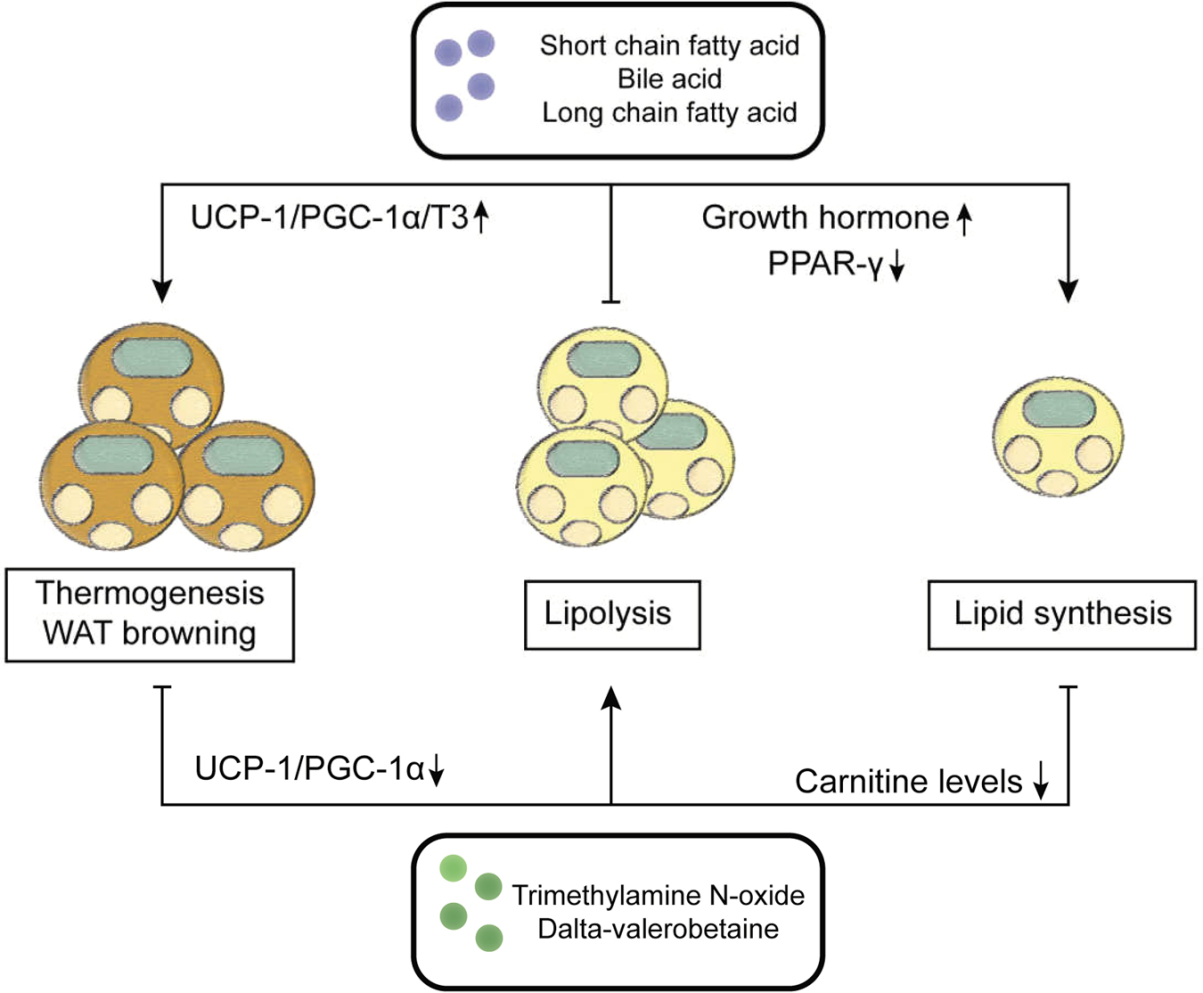
Metabolites affect adipose tissue function. Under the action of beneficial metabolites (short‐chain fatty acids [SCFAs], bile acids [BAs], long‐chain fatty acids [LCFAs]), adipose tissue promotes adipose thermogenesis and browning through uncoupling protein 1 (UCP‐1)/peroxisome proliferator‐activated receptor gamma‐coactivator 1α (PGC‐1α)/T3, promotes adipose decomposition through growth hormones produced by metabolites in the brain to balance energy metabolism in adipose tissue. In contrast, metabolites (trimethylamine N‐oxide [TMAO] and delta‐valerobetaine [VB]) inhibit browning and thermogenesis of adipose tissue, reduce lipid decomposition by reducing carnitine levels, and promote infiltration of immune cells and proinflammatory cytokines.

The imbalance of lipid decomposition and synthesis in adipose tissue is one of the factors leading to obesity and related diseases. In mouse 3T3‐L1 adipocytes, butyrate, and propionate have been shown to promote intracellular lipolysis by activating FFAR2/3.^123^ Butyrate supplements promote lipid oxidation by downregulating the expression and activity of PPAR‐γ.^34^ In addition, SCFAs also affect extracellular fat breakdown mediated by lipoprotein lipase (LPL).^124^ In vitro treatment of porcine preadipocytes with acetate, butyrate and propionate enhanced adipocyte differentiation, suggesting that SCFAs have lipogenic properties.^125^ This may be related to GPR43/FFAR2. In contrast, VB, a diet‐dependent obesogen, has been shown to promote tissue fat accumulation and reduce fatty acid utilization by reducing carnitine levels.^88^ Growth hormone has previously been reported to play an important role in the control of energy homeostasis by stimulating lipolysis and protein retention.^126^ SCFAs can also have positive effects by regulating growth hormone secretion and metabolism.^127^ Butyrate can stimulate growth hormone synthesis and promote growth hormone secretion induced by basal hormones and growth hormone‐releasing hormones. The results show that butyrate can improve lipolysis and oxidative metabolism (Figure 5).^128^

### 4.4 Affect chronic systemic inflammation

Systemic inflammation, characterized by an increase in the number of immune cells and tissue infiltration, is closely associated with insulin resistance and obesity.^129^ This is manifested by impaired intestinal barrier function and infiltration of inflammatory cells (CD4+ T cells, M1 macrophages, natural killer [NK] cells, and innate lymphocytes [ILCs]) into WAT, which promotes a proinflammatory state and leads to the development of the disease.

The establishment of a chronic inflammatory state and an increase in intestinal permeability are associated with an increase in lipopolysaccharide (LPS) in the host circulation. The establishment of chronic inflammatory states and increased intestinal permeability are associated with elevated levels of systemic LPS. This is well demonstrated in animal and human studies of obesity and related disease development.^130^ SCFAs, BAs, and indoles can enhance intestinal barrier function, reduce LPS circulation through the intestinal barrier, and reduce inflammation in the body. SCFAs (particularly butyrate) improve epithelial barrier function and intestinal permeability by regulating tight junction protein and mucin expression, and the reduced lumen pH adds an additional layer of protection against pathogen invasion and colonization.^131^ Tryptophan metabolism pathway changes in obese people are associated with systemic inflammation.^61^ IPA was shown to reduce the increase in high intestinal permeability and LPS in HFD‐fed mice.^71^ The mechanism is to improve intestinal permeability and inflammation by upregulating connexin‐coding mRNAs (ZO‐1, Occludin, and Claudin‐5).^64^, ^132^

These metabolites also have the ability to control the host immune response by regulating the differentiation, recruitment, and activation of neutrophils, dendritic cells (DCs), macrophages, monocytes, and T cells. First, neutrophils are important parts of the innate immune system. They reach the site of inflammation first and recruit other cells, including macrophages, by producing cytokines.^133^ SCFAs directly affect neutrophils and regulate the production of inflammatory cytokines. In vitro experiments reported that acetate and butyrate reduced the secretion of tumor necrosis factor‐α (TNF‐α) and nuclear factor κB (NF‐κB) from LPS‐stimulated neutrophils.^134^ In vitro supplementation of mice with acetate prevented the accumulation of neutrophils in the colon induced by a fiber‐free diet.^135^

Monocytes are large phagocytes which can turn into macrophages and DCs, and play a critical role in adaptive immune responses. SCFAs inhibit the maturation of monocytes, macrophages, and DCs, alter their ability to capture antigens, and reduce their production of proinflammatory cytokines (such as interleukin [IL]‐12 and TNF‐α). Similarly, BAs exert an anti‐inflammatory response through activation of TGR5 in macrophages, increasing intestinal IL‐10 levels^136^ and inhibiting the NF‐κB signaling pathway^137^ and NLRP3 inflammasome.^138^

**FIGURE 6:**
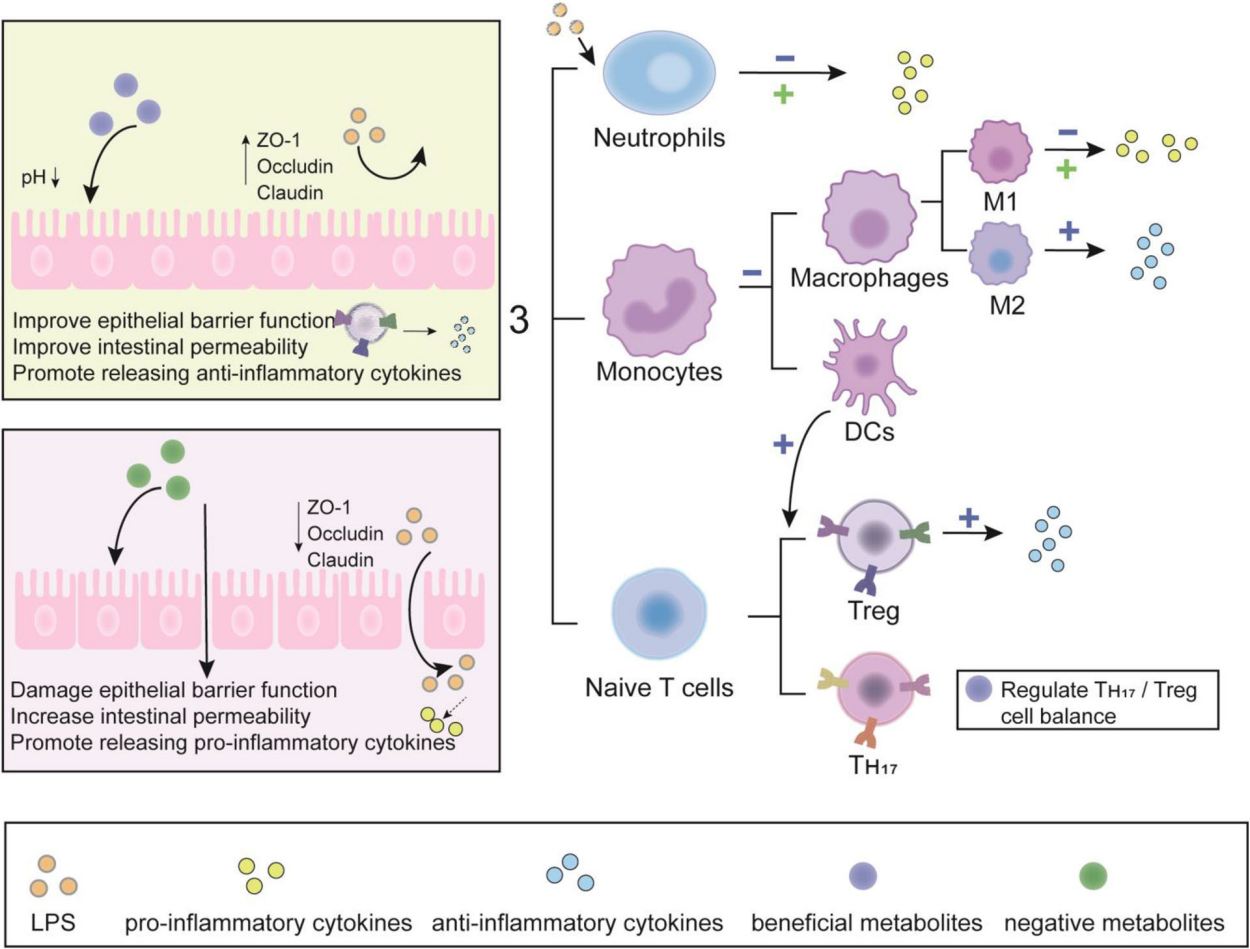
Metabolites affect chronic systemic inflammation. First, the beneficial metabolites in the intestinal tract decrease intestinal pH value and increase the expression of tight junction proteins (ZO‐1, Occludin, and Claudin), improve intestinal barrier and permeability, and reduce lipopolysaccharide (LPS) entry into the body. Metabolites then enter the body, preventing LPS‐stimulated neutrophil production of proinflammatory factors, reducing the maturation of macrophages and dendritic cells (DCs), or promoting the differentiation of M2 anti‐inflammatory cells. In addition, they are able to balance Treg/TH17 cells to regulate immunity while enhancing the ability of DCs to promote regulatory T (Treg) cell differentiation. Negative metabolites increase intestinal permeability and damage the intestinal barrier, and LPS easily enters the body, leading to systemic inflammation.

The number of regulatory T cells (Treg cells), which are important central regulators of antimicrobial immunity and tissue inflammation, is reduced in the visceral adipose tissue of obese individuals. SCFAs can rely on FFA2 to increase the number of Tregs and induce differentiation.^132^, ^139^ FFAR2 was found to increase the number of Tregs in the body.^140^ In addition to its direct promotion of Treg cell differentiation, butyrate enhanced the ability of DCs to promote Treg differentiation.^141^ BAs can regulate intestinal RORγ^+^ Treg cell homeostasis,^142^ increase Treg cell differentiation, and directly regulate the TH17/Treg cell balance.^143^ Indoles produced by intestinal flora metabolizing tryptophan can regulate miR‐181 in white adipocytes, increase Treg, eosinophils, ILCs and M2 macrophages, and inhibit M1 macrophages and TNF‐α, thereby regulating obesity.^144^ Because Indoles activate aromatic hydrocarbon receptors (AHRs), they regulate IL‐22 production by ILCs in the intestinal mucosa. While SCFAs promote IL‐22 production in CD4^+^ T cells and ILCs through histone deacetylase inhibition and GPR41.^145^, ^146^ Serum TMAO is thought to promote inflammation by increasing proinflammatory genes such as IL‐6 and chemokine ligands (Figure 6).^147^

## 5 CLINICAL APPLICATION OF GUT MICROBIOTA

Currently, therapeutic drugs and lifestyle interventions are recommended for obese and diabetic patients to alleviate metabolic problems such as obesity and diabetic. However, no matter the lifestyle intervention, medication or RYGB surgery, there are many problems such as poor efficacy, poor drug compliance, and other adverse reactions.^148^, ^149^, ^150^ Many previous animal and human studies have demonstrated the important role of intestinal flora in host energy metabolism. In addition to directly affecting various physiological functions of the host in the intestinal tract, the gut microbiota also indirectly affects the host circulation through metabolites. These studies provide new perspectives and feasible approaches for the application of intestinal flora in the treatment of obesity and related metabolic diseases: probiotics and prebiotics supplementation, FMT, and direct supplementation of the metabolites. More research is needed to confirm that using gut microbiota to treat obesity and related metabolic diseases appears to be a more personalized and safer approach than other therapies.^151^ Table 1 shows some examples of clinical applications.

**TABLE 1:** List of clinical application of gut microbiota

Clinical application	Study model	Effects in host related to obesity	Reference
*Akkermansia muciniphila*	Obese adults	Insulin sensitivity ↑ Insulinemia and total plasma cholesterol ↓	^5^
SCFAs mixtures	Normoglycemic men	Fasting fat oxidation, resting energy expenditure ↑ PYY ↑	^152^
Propionate	Overweight adult humans	PYY, GLP‐1 ↑ Energy intake and weight gain ↓	^30^
BAs	Participants with obesity and diabetes	Glucose, fructosamine, insulin, LDL, FGF19 ↓ GLP‐1, C‐peptide ↑	^153^
FMT	Obese adolescent	No effect of FMT on weight loss in adolescents with obesity	^154^
FMT	Adults with obesity and mild–moderate insulin resistance	No clinically significant metabolic effects during the study	^155^
FMT	Obese patients	Taurocholic acid levels↓ BAs profiles began to resemble No significant changes in mean BMI	^156^
FMT	Male with the metabolic syndrome	Not observe metabolic change insulin sensitivity ↑	^157^
FMT	Patients with severe obesity and metabolic syndrome	Improvements in HOMA2‐IR	^158^
FMT	Obese subjects with T2D	*Bifidobacterium* and *Lactobacillus* ↑ LDL cholesterol, liver stiffness ↓	^159^
Green‐Mediterranean dieters and FMT	Obese or dyslipidemic participants	Prevents weight gain and improves glucose tolerance	^160^

Abbreviations: BA, bile acid; BMI, body mass index; FGF, fibroblast growth factor; FMT, fecal microbiome transplantation; GLP‐1, glucagon‐like peptide 1; LDL, low‐density lipoprotein; SCFA, short‐chain fatty acid; T2D, type 2 diabetes.

The most common intervention for the gut microbiota is direct supplementation with specific probiotics and prebiotics. Long‐term consumption of fermented foods such as yogurt and cheese seems to be good for your health.^161^, ^162^ Well‐known probiotics such as *Bifidobacterium* and *L. plantarum* have been added to medicines and foods as bioactive substances to promote human health.^163^, ^164^ There have been many excellent reviews on this topic.^165^, ^166^, ^167^ It is worth mentioning that *A. muciniphila*, as a new generation of probiotics, has attracted much attention recently. In rodents, treatment with *A. muciniphila* reduces manifestations of obesity and improves metabolism, such as glucose tolerance, insulin resistance, steatosis, and intestinal permeability.^168^, ^169^ In clinical application, *A. muciniphila* also showed similar effects. After 6 weeks of diet intervention in obese and overweight adults, *A. muciniphila* was negatively associated with fasting blood glucose, waist‐to‐hip ratio, subcutaneous adipocyte diameter, and plasma triglycerides.^6^ More surprisingly, pasteurized *A. muciniphila* also improved insulin sensitivity, reduced insulinemia, and reduced plasma total cholesterol.^5^ This provides a new perspective for providing other probiotic agents.

Subsequently, the lab further demonstrated that *A. muciniphila* can play a beneficial role by activating PPAR‐α.^170^ However, there are not many clinical studies on *A. muciniphila*, and more independent studies are needed to provide evidence.

In recent years, studies on intestinal microbiota metabolites have mainly focused on their role as the “intermediate bridge” between gut microbiota and host, so there are few studies on the direct supplementation of metabolites in the treatment of obesity and related metabolic diseases in clinical studies. Most are concentrated in SCFAs and BAs. A study in obese and normal male populations using a mixture of SCFAs (including acetate, propionate, and butyrate) for rectal administration showed that SCFAs increased fat oxidation, resting energy expenditure, and PYY secretion in humans.^152^ Chambers et al.^30^ developed the inulin‐propionate ester for targeted delivery of propionate due to the poor adaptability of oral SCFAs in humans and its rapid absorption in the small intestine. The results showed a significant decrease in body weight, fat, and liver lipids, and prevented deterioration of insulin sensitivity. Another placebo‐controlled, double‐blind, randomized controlled trial using conjugated BAs sodium extract (IC‐CBAs) significantly reduced postprandial glucose, fasting insulin, fasting low‐density lipoprotein (LDL), and increased postprandial GLP‐1 levels.^153^ In addition, UDCA is commonly used to reduce symptomatic gallstone disease after RYGB.^171^, ^172^, ^173^ At present, this part of the research field is relatively blank, indicating that the research on new delivery system to alleviate and treat obesity and related diseases is still a little‐traveled road.

FMT is a means of transferring intestinal flora from host to host and has previously been shown to change obesity phenotype and metabolic function in many animal studies.^174^, ^175^, ^176^, ^177^ However, in several independent clinical trials, FMT appears to be controversial in improving body weight. Results of a randomized, double‐blind, placebo‐controlled trial of obese adolescents showed that FMT had no effect on weight loss in obese adolescents, despite a reduction in abdominal fat percentage. There was only a temporary improvement in glucose metabolism and insulin sensitivity at 6 weeks.^154^ Previous clinical trials have also found that FMT appears to have limited effects on weight and metabolism.^155^, ^156^, ^157^ However, a randomized clinical trial of FMT and fiber supplementation in patients with severe obesity and metabolic syndrome found significant improvements in subjects' insulin sensitivity.^158^ FMT and lifestyle intervention increased the abundance of *Bifidobacterium* and *Lactobacillus*, and significantly improved the blood lipid and liver hardness of the subjects.^159^ Similarly, FMT can be combined with a Mediterranean diet and foods like green tea to maintain weight and stabilize blood sugar.^160^ This may be because transplanting gut microbiota alone is difficult to produce a stable effect in the subjects’ intestines, and combined with other methods that facilitate probiotic colonization, FMT could significantly improve obesity and metabolism.

## 6 DISCUSSION AND CONCLUSION

Previous animal and human studies have highlighted the importance of gut microbiota in regulating host metabolic processes. But there are still some problems. First, are gut microbiota and host bidirectional regulation? Are certain intestinal microbiota metabolites circulating in the host a marker or a consequence of impaired metabolism? ImP, for example, is found at higher levels in the blood of T2D patients and is associated with inflammation, while diet also alters ImP levels by altering gut microbiota. Thus, ImP levels may be a sign of impaired metabolism and, in turn, may contribute to the development of disease. Second, the effect of gut microbiota metabolites on the host is sometimes contradictory in various animal and human studies. Butyrate is generally thought to improve metabolic function. However, one study found that butyrate was associated with increased intramural fat deposition and insulin resistance in the offspring of pregnant female SD rats.^35^ Diet, exercise, and a variety of other factors can affect the composition and structure of the gut microbiota. Changes in the gut microbiota, in turn, affect the production of metabolites and subsequently alter a variety of complex physiological activities in the body, as well as the competition/synergy between microbes. Thus, the host–microbe axis can be bidirectional and complex. Furthermore, the mechanism of gut microbiota metabolites has been verified in rodents, but the composition and structure of the human intestinal microbiota are different from those of rodents. Clinical trials of gut microbiota are based on animal studies, but do not necessarily yield similar results. Therefore, it needs to be explored further.

Research in the field of the gut microbiota over the past two decades has shown that human biology is particularly relevant to coexisting microorganisms, most of which live in the digestive tract, where they produce or modify a variety of chemicals that directly or indirectly participate in and influence a variety of physiological functions, including energy metabolism and immune and neurological functions. Although this article cannot cover all aspects of current gut microbiota research, it is certain that substances produced by gut microbiota metabolic activities in the gut will have clinical significance in the future treatment of obesity and related metabolic diseases. We also have some challenges. First, regulating gut microbiota can be used to treat metabolic diseases clinically, because it seems safer and more personalized than other approaches. However, as mentioned in Section 5, the clinical effects of FMT are not satisfactory. It seems that the effects of obesity alleviation and metabolism improvement are not so significant. When combined with other treatments, such as cellulose blends or lifestyle interventions, results are better. Therefore, more stable methods are needed to regulate the gut microbiota. Second, the applications of a new generation of probiotics are what we want. *A. muciniphila* is a new generation of probiotics. Although many animal experiments have proved its beneficial effects and elaborated its mechanism, few clinical trials have been carried out at present. However pasteurization of *A. muciniphila* offers a new perspective on new forms of treatment.^5^ In addition, the gut microbiota can be used as an adjunct to drug therapy. As clinical trials have demonstrated that probiotics alone can improve glucose homeostasis.^178^ Single or multiple probiotics can also assist metformin or other diabetes drugs to treat T2D and improve fasting glucose, insulin resistance and the intestinal barrier.^25^, ^179^ It can be used as an adjunct to drugs and can be tailored to individual patients. And last, gut microbiota metabolites can also be used as a drug, and new agents can be developed to deliver intestinal microbiota metabolites for better absorption or targeting.

Finally, the gut microbiota is far more complex than previously known. The gut microbiota goes far beyond bacteria, including fungi, phages, and eukaryotic viruses. They compete and collaborate with each other. Moreover, it is important that the large number of active substances produced by gut microbiota affect host physiology and pathology, providing new ideas for the prevention or treatment of common metabolic disorders.

## AUTHOR CONTRIBUTIONS

K.L. conceived the concept of the review article and wrote the sections. L.Z. screened and sorted out the references. L.Y. supervised the writing and final revision of the manuscript. All authors have read and agreed to the published version of the manuscript.

## CONFLICT OF INTEREST

The authors declare they have no conflicts of interest.

## ETHICS STATEMENT

Not applicable.

## Data Availability

Not applicable.
